# Editorial: Tumor metabolism: From molecular mechanisms to clinical application

**DOI:** 10.3389/fcell.2023.1158467

**Published:** 2023-02-17

**Authors:** Chunming Cheng, Si Zhang, Dongyin Guan, Yi Wang, Nidhi Jyotsana

**Affiliations:** ^1^ Department of Radiation Oncology, Ohio State Comprehensive Cancer Center, College of Medicine at The Ohio State University, Columbus, OH, United States; ^2^ Department of Biochemistry and Molecular Biology, NHC Key Laboratory of Glycoconjugates Research, School of Basic Medical Sciences, Fudan University, Shanghai, China; ^3^ Division of Diabetes, Endocrinology, and Metabolism, Department of Medicine, Baylor College of Medicine, Houston, TX, United States; ^4^ Department of Critical Care Medicine, Sichuan Academy of Medical Science and Sichuan Provincial People’s Hospital, University of Electronic Science and Technology of China, Chengdu, China; ^5^ Department of Cell and Developmental Biology, Vanderbilt University, Nashville, TN, United States

**Keywords:** tumor, metabolism, mechanism, clinical application, metabolic pathways, therapeutic strategies

Tumors utilize abnormal metabolic activities to support cancer growth in different organ environments. Tumor metabolism is becoming a hot topic within cancer research, with the possibility to impact our approaches to tumor detection and treatment in the future, to improve cancer patient outcomes. However, to achieve these goals, we need to improve our understanding and knowledge in the field of tumor metabolism. This includes understanding the molecular mechanisms in the tumor microenvironment (TME), finding effective gene targets, and deciphering the role of these targets in specific organ environments. In this research topic “Tumor Metabolism: from Molecular Mechanisms to Clinical Application” a Research Topic of six papers, including high-quality original research articles, brief research reports, and reviews, have been accepted to address novel metabolic regulatory mechanisms, new detection technologies in TME, potential translational applications (e.g., cancer screening and diagnosis) and therapeutic strategies, recent advances and the impact of tumor metabolism on cancer therapy.

The metabolic control in the native tumor micro-environment. We currently lack a complete and deep understanding of the native TME, especially how to directly detect and control its nutrient and effect on different cell types, including immune cells and tumor cells. The breakthrough in this direction may profoundly improve cancer treatment. Valvo et al. from Harvard University apply novel implantable microdevices to *in situ* tumors to perform a systematic functional investigation into the metabolic regulations between infiltrated immune cells and tumor cells in the TME. It is an *in situ* competition assay between malignant and immune cells within tumors using a range of localized micro-dose metabolic perturbations. This approach may be used to investigate the effects of various metabolites or inhibitors on *in situ* tumor growth by targeting the enriched metabolites or depletion of specific metabolites.

*The rapid screening and diagnosis of cancer, based on tumor metabolic fingerprinting.* The sensitivity, specificity, and cost of early metabolite detection in cancer restricts its translational application. Therefore, early screening and diagnosis of cancer based on tumor metabolic fingerprinting becomes a very important clinical area. Song et al. from Fudan University provide a fast and easy approach for the rapid screening and diagnosis of triple-negative breast cancer (TNBC). They successfully utilize mass spectrometry (MS)-based metabolic fingerprinting on the biological specimens available through biopsy for TNBC screening and diagnosis along with the validation of the sensitivity and specificity of this method. This may establish a cost-effective pipeline for early screening and diagnosis of various cancer types in the clinic.

*The anti-tumor mechanism of gluconeogenesis mediated by NFYAv2.* In the TME, gluconeogenesis not only inhibits aerobic glycolysis but it can also interfere with other metabolic pathways. Elucidating the regulation of key gluconeogenic enzymes may benefit cancer treatment. Tsujimoto et al. from Okayama University, Japan, find that the upregulation of NFYAv2, one of NFYA isoforms, induced by glucose deprivation promotes the gluconeogenic enzyme PCK1 transcription leading to high reactive oxygen species (ROS) and cell death in hepatocellular carcinoma (HCC). This finding may help us to develop potential anti-tumor therapies by augmenting the NFYAv2-gluconeogenesis axis in HCC.

*Immunometabolic dysfunction contributing to obesity-induced HCC.* Non-alcoholic steatohepatitis (NASH), the obesity-associated fatty liver disease, has been associated with the development of HCC. In addition, immunometabolic dysfunction is a major characteristic of NASH. Akl et al. discuss the relationship between obesity and liver cancer, the underlying drivers of disease progression and metabolic adaptation, and obesity-linked alterations in tumor immunity. A clear and full understanding of obesity-associated immune-metabolic changes may improve options for the HCC treatment.

*A predictive model for the glycolysis-related poor prognosis in diffuse large B-cell lymphoma (DLBCL).* Abnormal glycolysis is the key characterization of malignancy. In DLBCL, the most aggressive and malignant subtype of lymphoma, Cui et al. construct a model using glycolysis-related genes for risk stratification, prognosis prediction, and immune landscape evaluation. Their predictive model reveals that increased glycolysis of DLBCL might be an indicator of the malignancy of these patients due to the suppressed lymphocyte reaction and increased tumor immune escape controlled by immune checkpoints. Accurate risk identification of cancer patients is the key to prognosis and treatment decision-making.

*Proton-coupled monocarboxylate transporters (MCTs)-mediated lactate signaling and effect in tumor therapy.* Tumor aerobic glycolysis leads to an accumulation of lactic acid and the acidification of the tumor microenvironment, which may seriously affect cancer therapy. To face these challenges, Duan et al. comprehensively reviewed the roles of MCTs-mediated lactate signaling in cancer and discuss its effect on tumor treatment. The study will further deepen the understanding of lactate signaling and provide new anti-tumor treatment options.

Taken together, tumor metabolism research is developing rapidly. We understand that the development of new technologies in this field and tumor metabolism-based clinical applications will be the most important direction. Due to time limitations, we only collected six manuscripts here on the research topic of tumor metabolism. We hope that these studies can provide some insights into the development of tumor metabolism and benefit cancer therapy.

